# Receptor profiling and growth assessment of influenza A virus in porcine mammary and non-mammary tissues and derived cells

**DOI:** 10.1128/jvi.00615-26

**Published:** 2026-07-06

**Authors:** Ulises Barron-Castillo, Nathalie Berube, Cynthia L. Swan, M. Afzal Javed, Lauren Aubrey, Jill Trann, Makenzie Gidych, Sauhard Shrivastava, Kaushal Baid, Arinjay Banerjee, Yan Zhou

**Affiliations:** 1 Vaccine and Infectious Disease Organization (VIDO), University of Saskatchewan 7235, Saskatoon, Saskatchewan, Canada; 2 Department of Veterinary Microbiology, Western College of Veterinary Medicine, University of Saskatchewan 7235, Saskatoon, Saskatchewan, Canada; University of Freiburg, Freiburg, Germany

**Keywords:** HPAI, mammary gland, respiratory tract, sialic acid receptor, virus binding

## Abstract

Highly pathogenic avian influenza (HPAI) virus clade 2.3.4.4b genotype B3.13 infected the mammary gland of dairy cattle; the new tissue tropism and host heightened concern about its ability to cross species barriers with zoonotic potential. Pigs play a key role in influenza A virus (IAV) adaptation, serving as a “mixing vessel” for the emergence of reassortants. The susceptibility of porcine mammary gland to HPAI infection remains unexplored. In this study, we profiled IAV receptors in porcine mammary gland as well as respiratory tract tissues. Additionally, we evaluated the binding capacity of IAVs to these tissues. Furthermore, we isolated primary cells from porcine mammary gland and respiratory tract, and immortalized them. We examined the growth potential of IAV isolates from bovine, avian, swine, and human on these cells. We showed that porcine mammary gland displays both SA-α2,3 and SA-α2,6, and that IAVs bind to mammary gland tissues with variable affinities. While bovine H5N1 virus replicates efficiently in mammary gland and respiratory tract cells, replication of other IAVs in mammary epithelial cells is moderate but is efficient in respiratory cells. These findings suggest that porcine mammary gland could support the infection by HPAI 2.3.4.4b genotype B3.13.

**IMPORTANCE:**

Our findings expand the current understanding of IAV tissue tropism in swine by revealing that the porcine mammary gland may serve as an alternative and previously overlooked replication site for both avian and mammalian-origin viruses. Given the increasing frequency of interspecies spillover events and expanding host range of HPAI H5N1, our finding that mammary epithelial cells have an intrinsic capacity to support replication of diverse IAV strains provides new insight into the porcine mammary gland as a potential replication and reassortment site for IAV.

## INTRODUCTION

The recent isolation of highly pathogenic avian influenza (HPAI) H5N1 clade 2.3.4.4b genotype B3.13 and D1.1 in the mammary gland of dairy cattle revealed a novel tissue tropism and raised concerns regarding viral specie-adaptation. While spillover events from birds to mammals have been previously documented, the emergence of HPAI in dairy cattle suggests an expansion of host range and potential alterations in receptor binding dynamics (1–3). One of the key determinants of influenza A virus (IAV) host specificity and tissue tropism is the virus’ ability to bind to sialic acid (SA) receptors on the surface of host cells (4, 5). Avian IAV, including HPAI H5N1, preferentially binds to SA-α2,3 receptors, which are abundantly expressed in the avian respiratory and gastrointestinal tracts, and are also expressed in the lower respiratory tract of humans (6, 7). In contrast, human IAV typically binds to SA-α2,6, which is predominantly expressed in the upper respiratory tract of both humans and swine (8, 9). Various studies have examined and compared the distribution of SA receptors in bovine respiratory tract and mammary gland tissues, showing that both tissues express both SA-α2,3 and SA-α2,6 receptors (10). The expression of SA-α2,3 receptors for avian hemagglutinins (HA) in bovine mammary gland provides a rationale for susceptibility of this tissue to HPAI H5N1 infection.

During the dairy cattle H5N1 spillover, concurrent cases of infections were documented in domestic cats on affected farms. These infected cats showed systemic infection with widespread lesions and elevated viral RNA in the brain. Lectin histochemistry revealed co-expression of SA receptors in brain and other tissues, highlighting a potential risk of neuro-invasion for HPAI H5N1 (11). A recent study evaluated the binding specificity of bovine H5N1 HA to human tissues, including pulmonary, mammary, and conjunctival tissues (12). While bovine H5N1 HA can bind slightly to SA-α2,6 receptor, it binds preferentially to SA-α2,3 receptor. Moreover, bovine H5N1 HA binds effectively to multiple human tissues including conjunctiva, mammary gland, trachea, and lungs, indicating broad tissue tropism (12). These findings suggest that the H5N1 B3.13 strain has evolved the capacity to bind to multiple tissue types from different mammalian species. Its ability to replicate in organs that are not typical organ targets, including the mammary gland, brain, and conjunctiva, underscores the need to investigate receptor distribution and viral replication in other species beyond the respiratory tract.

Pigs are known as mixing vessels for IAVs due to the presence of both SA-α2,3 and SA-α2,6 receptors in their respiratory tract, allowing co-infection by avian, human, and swine IAV strains (13, 14). The expression of both receptors facilitates viral reassortment, enabling the emergence of novel viruses with pandemic potential, such as the 2009 H1N1, a quadruple-reassortant strain (15). Given the recent evidence of HPAI H5N1 replication in bovine mammary gland tissue, it is important to assess whether the porcine mammary gland could similarly support influenza viral infection. This will provide insights into whether it serves as a potential site for viral adaptation and reassortment. Meanwhile, it is also equally important to assess the potential replication of bovine H5N1 in porcine respiratory tract in comparison to other IAV strains. Here, we characterized IAV receptors on porcine mammary gland and respiratory tract tissues. Additionally, we tested the binding capacity of IAV to these tissues. Furthermore, we isolated primary cells from both porcine mammary gland and respiratory tract tissues and immortalized them. The growth potential of a panel of IAV isolates from bovine, swine, human, and avian sources was examined in these cells. We report that porcine mammary gland expresses both SA-α2,3 and SA-α2,6 receptors, and IAVs bind to mammary gland tissues with variable levels. HPAI H5N1 bovine isolate replicates efficiently in epithelial cells derived from the mammary gland and respiratory tract. In contrast, non-HPAI isolates exhibited moderate replication in mammary gland cells but efficient replication in respiratory tract cells. These findings suggest that porcine mammary gland tissues could support infection by HPAI H5N1 clade 2.3.4.4b genotype B3.13.

## RESULTS

### Porcine mammary gland and respiratory tract tissues display both SA-α2,3 and SA-α2,6 receptors

We first characterized the distribution of SA receptors in mammary gland and respiratory tract tissues (nasal turbinate, trachea, bronchiole, and alveoli), which are the primary targets for IAV infection. The expression of SA receptors was detected by using lectins: MAL-I, MAL-II, and SNA. MAL-I binds to carbohydrate chains containing SA residues linked α2,3 to galactose residues and linked to β1-4 N-acetylglucosamine ([GlcNAc] SA-α2,3-Gal-β1,4-GlcNac), MAL-II binds to carbohydrate chains SA-α2,3-Gal-β1,3-GlcNac (16), and SNA is a lectin that binds preferentially to SA attached to Gal or GalNAc at α−2,6 (SA-α2,6-Gal/GalNAc) (17). As negative controls, tissue slides were treated with sialidase prior to lectin staining.

**Fig 1:**
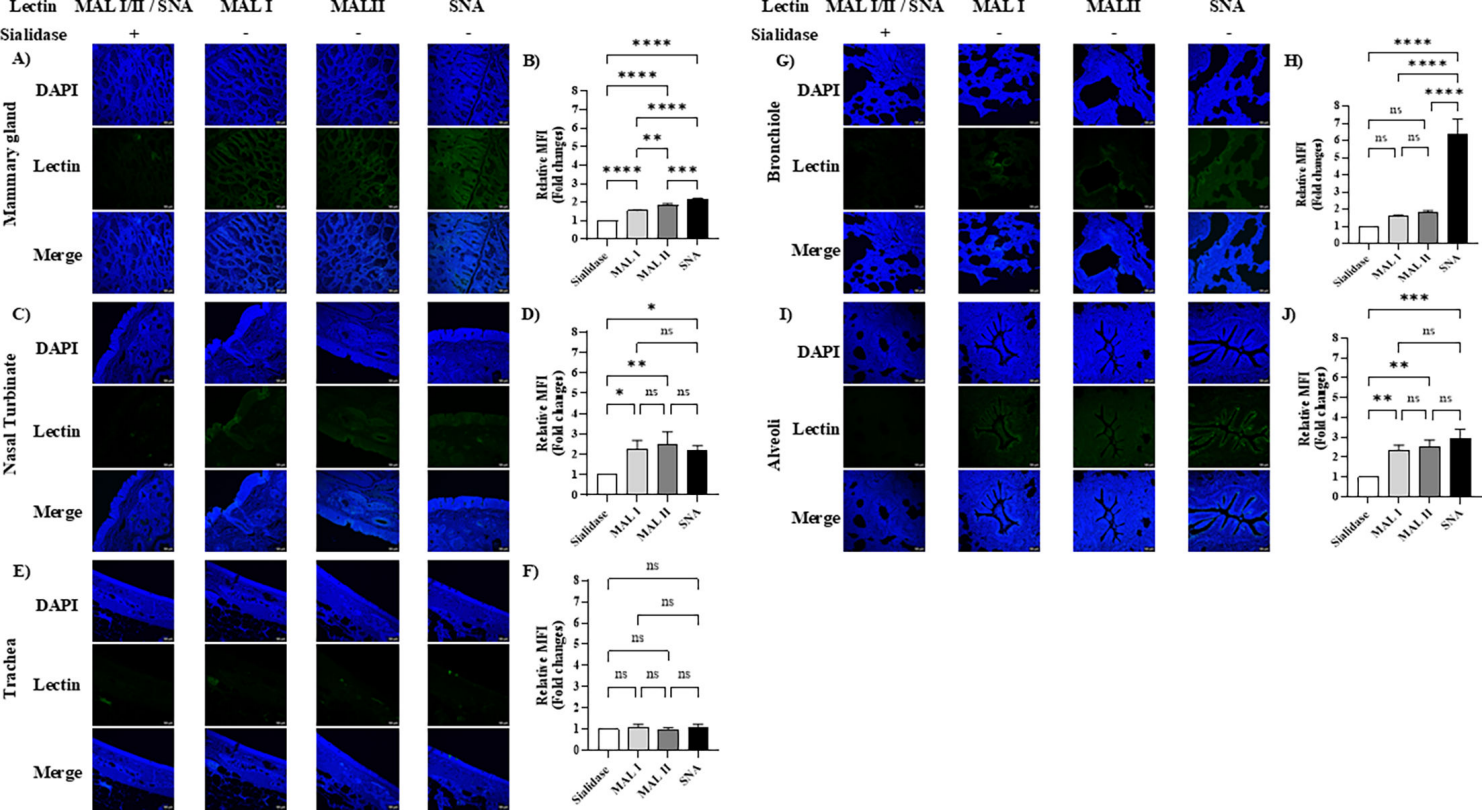
Expression of SA-α2,3 and SA-α2,6 receptors in porcine tissues. Mammary gland (**A**), nasal turbinate (**C**), trachea (**E**), bronchiole (**G**), and alveoli tissue (**I**) sections were stained with biotinylated MAL-I (SA-α2,3-Gal-β1,4), MAL-II (SA-α2,3-Gal-β1,3), and SNA (SA-α2,6), followed by incubation with Streptavidin-Alexa Fluor 488 (green) and DAPI (blue). Sialidase +: Tissue sections were treated with sialidase A prior to lectin staining. The lectin expression levels in mammary gland (**B**), nasal turbinate (**D**), trachea (**F**), bronchiole (**H**), and alveoli (**J**) were quantified by relative fold change of MFI calculated from three different areas of interest. Error bars represent standard deviation. One-way ANOVA was used to compare the lectin levels. ns indicates non-significant; * indicates *P* < 0.05; ** indicates *P* < 0.01; *** indicates *P* < 0.005; **** indicates *P* < 0.001. Scale bar = 50 µm.

As shown in Fig. 1A, we observed that the porcine mammary gland expressed both SA-α2,3 and SA-α2,6 receptors. While the expression of SA-α2,3-Gal-β1,4 and SA-α2,3-Gal-β1,3 in the mammary gland was detected mainly in the epithelium, SA-α2,6 was observed in the epithelium and lumen. Receptor expression levels were quantified by first normalizing the mean fluorescence intensity (MFI) to the DAPI MFI within the same sample, and then expressing the values as the relative fold change compared with sialidase-treated samples. Mammary gland expressed significantly higher levels of SA-α2,6 receptor (2.18-fold change), SA-α2,3-Gal-β1,4 (1.54-fold), and SA-α2,3-Gal-β1,3 (1.82-fold change) receptors (Fig. 1B). Regarding nasal turbinate tissue, SA-α2,3-Gal-β1,4 and SA-α2,3-Gal-β1,3 receptors were detected in epithelium, sub-epithelium, and mucous gland, whereas the SA-α2,6 receptor was detected mainly in epithelium and sub-epithelium, with similar levels of relative MFI ranging between 2.22- and 2.50-fold change (Fig. 1C and D). Trachea expressed very low levels of SA receptors, with 1.13-fold change in relative MFI for SA-α2,6, 1.08-fold change for SA-α2,3-Gal-β1,4, and 0.99-fold change for SA-α2,3-Gal-β1,3 (Fig. 1E and F). In bronchiole, significant high levels of SA-α2,6 were detected, showing 6.36-fold change in MFI compared with the sialidase-treated tissues, whereas SA-α2,3-Gal-β1,4 and SA-α2,3-Gal-β1,3 showed moderate levels of expression (1.58- and 1.80-fold change of MFI, respectively) (Fig. 1G and H). Alveoli tissue displayed both SA-α2,3 and SA-α2,6 receptors in the epithelium; SA-α2,6 expression showed a 2.93-fold increase in relative MFI, and SA-α2,3-Gal-β1,4 and SA-α2,3-Gal-β1,3 showed 2.35- and 2.45-fold change, respectively (Fig. 1I and J).

Overall, the expression of SA-α2,3 and SA-α2,6 receptors was detected across all tissues, though at various levels. The highest expression of SA-α2,6 was detected in bronchiole and alveoli tissues, while the highest expression of SA-α2,3 was observed in nasal turbinate and alveoli. Of note, trachea exhibits the lowest expression of both types of receptors.

### Bovine, avian, and swine IAVs bind to porcine mammary gland, bronchiole, and alveoli tissues

To determine whether IAV isolates from various species were able to bind to the SA receptors displayed in the mammary gland and respiratory tissues, porcine tissue sections were incubated with inactivated bovine H5N1, low pathogenic avian influenza (LPAI) H5N1 (referred to as avian H5N1 hereafter), or swine H1N2 viruses, and the binding was detected via immunofluorescent staining. To verify SA-dependent binding, porcine tissues were treated with sialidase prior to incubation with virus and were used as negative controls.

**Fig 2:**
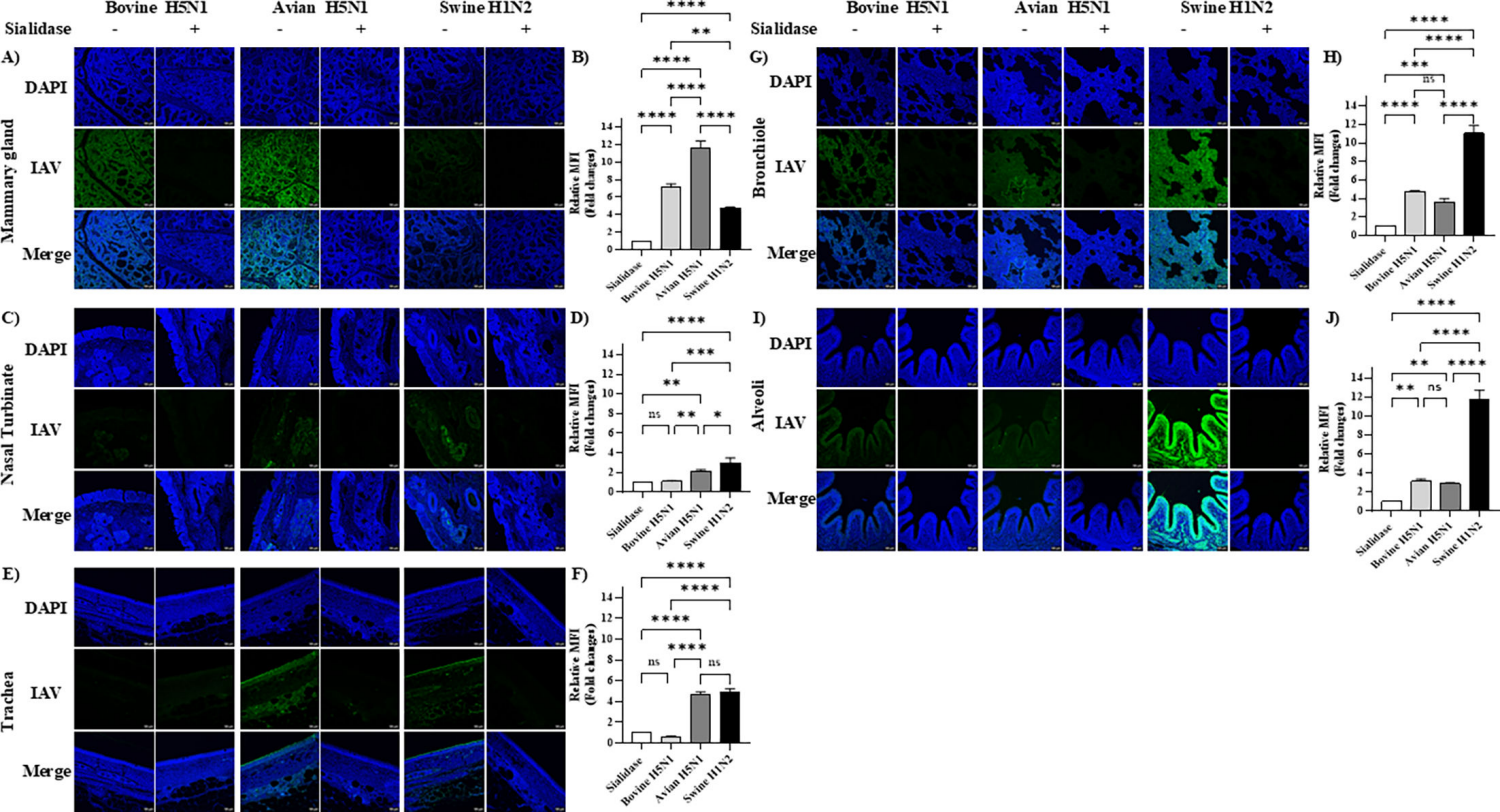
Binding of bovine H5N1, avian H5N1, and swine H1N2 to porcine tissues. Porcine mammary gland (**A**), nasal turbinate (**C**), trachea (**E**), bronchiole (**G**), and alveoli tissue (**I**) sections were incubated with purified and inactivated bovine H5N1, avian H5N1 virus, or swine H1N2 virus. The attached bovine H5N1, avian H5N1, and swine H1N2 viruses were detected using rabbit polyclonal anti-M1 antibody. Subsequently, all sections were stained with Alexa Fluor 488 conjugated anti-rabbit IgG H&L (green) and DAPI (blue). Control tissue sections were treated prior to virus incubation with sialidase A to assess receptor-specific interactions (Sialidase +). The binding levels in mammary gland (**B**), nasal turbinate (**D**), trachea (**F**), bronchiole (**H**), and alveoli (**J**) were quantified by relative MFI from three different areas of interest. One-way ANOVA was used to compare the binding levels. ns indicates non-significant; * indicates *P* < 0.05; ** indicates *P* < 0.01; *** indicates *P* < 0.005; **** indicates *P* < 0.001. Scale bar = 50 µm.

In mammary gland tissue, bovine H5N1 and avian H5N1 viruses showed robust binding (7.11- and 11.59-fold change in MFI, respectively), whereas swine H1N2 virus exhibited moderate binding (4.77-fold change in MFI) (Fig. 2A and B). In nasal turbinate, binding of avian H5N1 (2.15-fold change) and swine H1N2 (2.99-fold change) was observed; however, binding of bovine H5N1 was weak (1.10-fold change) (Fig. 2C and D). In the trachea, moderate binding of avian H5N1 and swine H1N2 (4.65- and 4.92-fold change, respectively) was observed, yet bovine H5N1 showed no binding (Fig. 2E and F). In bronchioles, binding of bovine H5N1 and avian H5N1 was moderate (4.79- and 3.62-fold changes, respectively) (Fig. 2G and H). Similar levels were observed in alveoli for these two viruses (3.14- and 2.90-fold changes) (Fig. 2I and J). Notably, swine H1N2 displayed significantly higher binding in both bronchioles and alveoli (11.06- and 11.77-fold changes, respectively).

### Porcine primary epithelial cells express both SA-α2,3 and SA-α2,6

**Fig 3:**
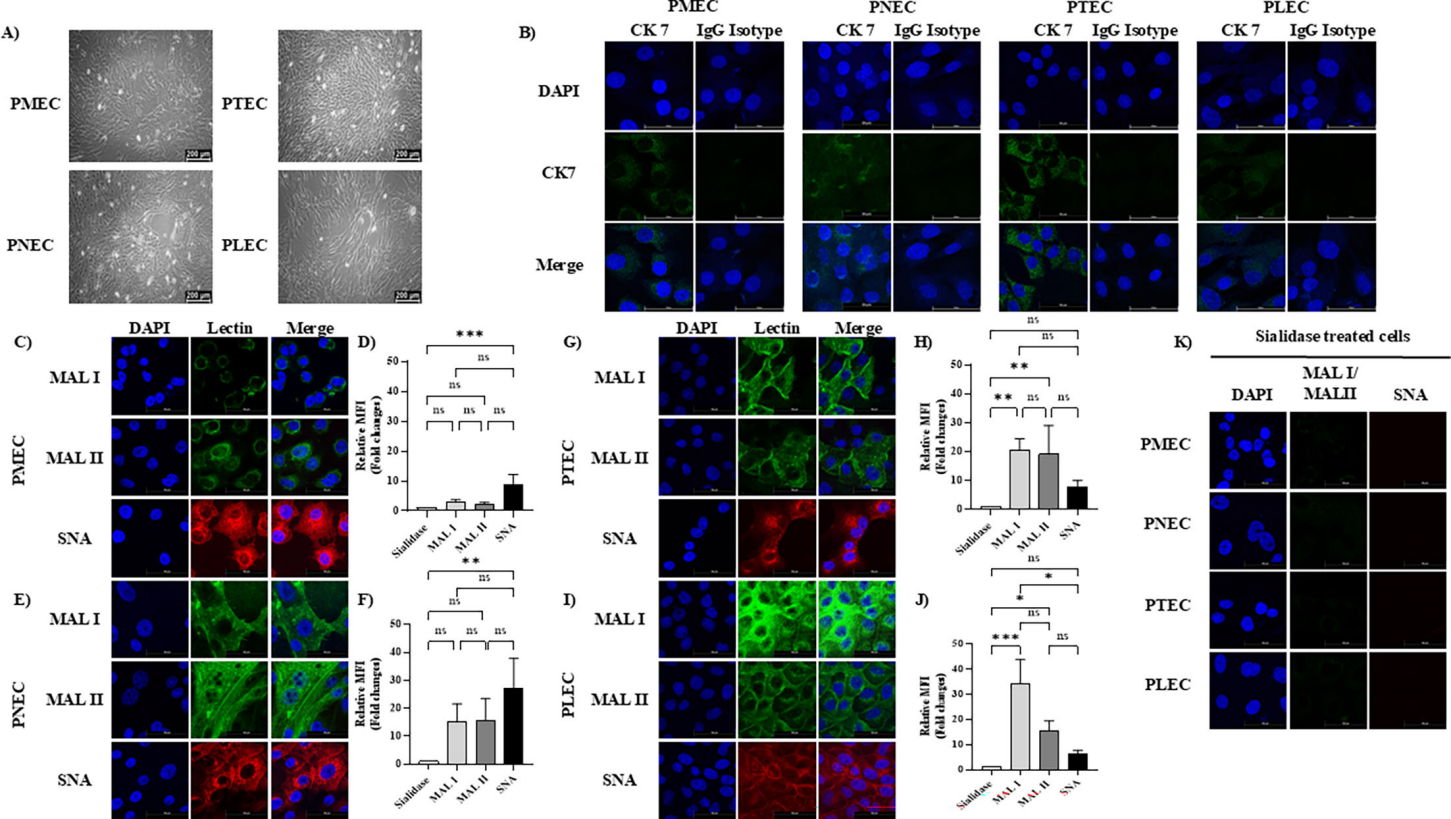
Expression of SA-α2,6 and SA-α2,3 receptors in porcine primary epithelial cells. (**A**) Morphology of primary mammary gland (PMEC), porcine nasal turbinate epithelial cells (PNEC), trachea (PTEC), lung (PLEC). Scale bars = 200 µm. (**B**) Identity of epithelial cells by detection of the epithelial marker cytokeratin-7. Porcine primary epithelial cells were stained with either monoclonal anti-CK7 or mouse IgG isotype, followed by staining with anti-mouse IgG H&L (Alexa Fluor 488, green) and DAPI (blue). Porcine primary mammary gland (**C**), nasal turbinate (**E**), trachea (**G**), and lung (**I**) cells were stained with biotinylated MAL-I (SA-α2,3-Gal-β1,4), MAL-II (SA-α2,3-Gal-β1,3), or SNA-Cy5 (SA-α2,6) (red). Cells stained with MAL-I or MAL-II were then stained with Streptavidin-Alexa Fluor 488 (green). Subsequently, all slides were stained with DAPI (blue). Porcine primary epithelial cells were treated with sialidase prior to lectin staining were used as control (**K**). The lectin expression levels in porcine primary mammary gland (**D**), nasal turbinate (**F**), trachea (**H**), and lung (**J**) cells were quantified by MFI from three areas of interest. Kruskal-Wallis test was used to compare the lectin levels. ns indicates non-significant; * indicates *P* < 0.05; ** indicates *P* < 0.01; *** indicates *P* < 0.005. Scale bar = 50 µm.

Our findings thus far suggest that the porcine mammary gland may be susceptible to infection by various IAV strains. To further investigate this potential, we isolated epithelial cells from the porcine mammary gland, nasal turbinate, trachea, and lung (alveoli). We assessed the capacity of porcine epithelial cells to support replication of various IAVs. Epithelial cells were dissociated from tissues with collagenase and hyaluronidase. Nasal turbinate and trachea cells showed a cobblestone-like morphology, which is a characteristic of epithelial cells. In contrast, lung and mammary gland cells showed more elongated morphology (Fig. 3A).

To confirm epithelial cell identity, immunofluorescence staining was performed using an antibody against cytokeratin-7 (anti-CK7), a well-established epithelial marker. Confocal microscopy revealed cytoplasmic staining in all cells (Fig. 3B), confirming the isolated cells from all tissues were of epithelial origin. Then, we examined the expression of SA-α2,3 and SA-α2,6 in these cells using MAL-I, MAL-II, and SNA lectins. All cell types expressed both types of SA receptor, though distinct levels of expression were observed for each tissue. Porcine primary mammary gland epithelial cells (PMEC) expressed predominantly SA-α2,6 receptors (8.78-fold change), but also expressed moderate levels of SA-α2,3-Gal-β1,4 (2.80-fold change) and SA-α2,3-Gal-β1,3 (2.23-fold change) receptors (Fig. 3C and D). Porcine primary nasal turbinate epithelial cells (PNECs) displayed significantly high levels of SA-α2,6 (27.23-fold change in MFI), followed by the expression of SA-α2,3-Gal-β1,4 and SA-α2,3-Gal-β1,3 (15.05- and 15.53-fold change, respectively) (Fig. 3E and F). Porcine primary trachea epithelial cells (PTECs) expressed significantly high levels of SA-α2,3-Gal-β1,4 and SA-α2,3-Gal-β1,3 (20.4- and 19.06-fold change, respectively) and moderate level of SA-α2,6 (7.83-fold change) (Fig. 3G and H). Porcine lung epithelial cells (PLECs) expressed significantly high levels of SA-α2,3-Gal-β1,4 (34.30-fold change) and SA-α2,3-Gal-β1,3 (15.42-fold change), and moderate level of SA-α2,6 (6.62-fold change), which was statistically different (Fig. 3I and J). Sialidase-treated cells were stained negatively for MAL-I, MAL-II, and SNA (Fig. 3K).

### Porcine primary mammary gland epithelial cells support replication of swine, avian, and human-origin IAVs at different degrees

We were able to show that porcine epithelial cells from different tissues express SA receptors. Therefore, we next evaluated the replication of the bovine H5N1 virus in porcine cells in comparison to other IAVs known to replicate in swine respiratory tract. Primary nasal turbinate, trachea, lung and mammary gland epithelial cells were infected with bovine H5N1, avian H5N1, swine H1N2 or human H1N1 at multiplicity of infection (MOI) of 0.001, and viral titer was determined at indicated time points.

**Fig 4:**
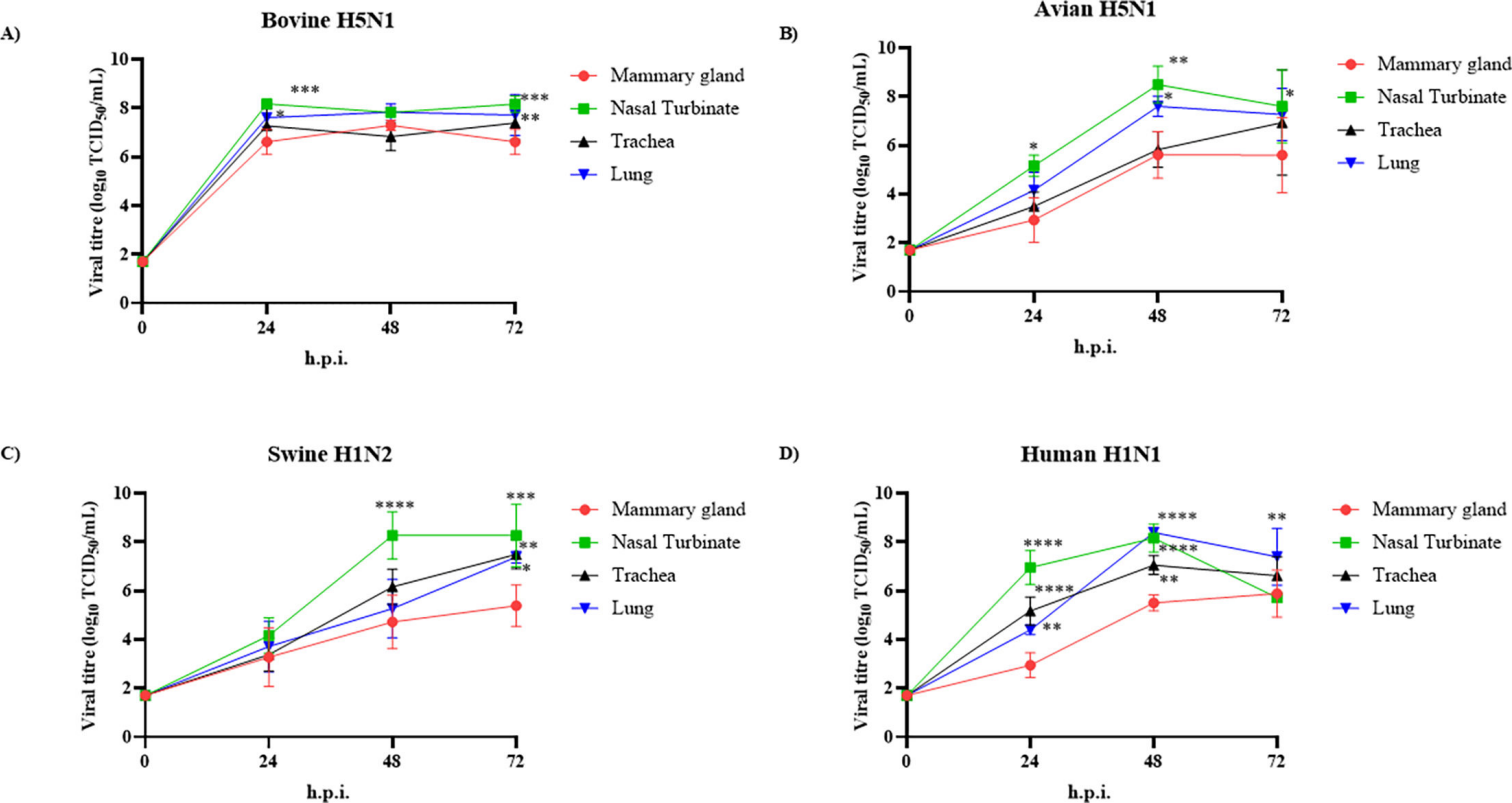
Replication kinetics of bovine, avian, swine, and human IAVs in porcine primary epithelial cells. Primary epithelial cells isolated from the mammary gland, nasal turbinate, trachea, and lung were infected with bovine H5N1 (**A**), avian H5N1 (**B**), swine H1N2 (**C**), and human H1N1 (**D**) at MOI of 0.001. Supernatant was harvested at 24, 48, and 72 hpi, and titrated on MDCK cells by TCID_50_ assay. Data represent the mean of three independent experiments; error bars indicate the standard deviation. Significance was calculated by two-way ANOVA; * indicates *P* < 0.05; ** indicates *P* < 0.01; *** indicates *P* < 0.005; **** indicates *P* < 0.001.

Bovine H5N1 virus replicated efficiently and rapidly across all epithelial cell types, reaching plateau at 24 hpi (Fig. 4A). Viral titers reached 8.18 log_10_ TCID_50_/mL in nasal epithelial cells and 6.62 log_10_ TCID_50_/mL in mammary gland epithelial cells. Avian H5N1 virus replicated steadily, with increasing titers, and peaked at 48 hpi in the nasal turbinate (8.49 log_10_ TCID_50_/mL), lung (7.61 log_10_ TCID_50_/mL), and mammary gland (5.83 log_10_TCID_50_/mL) epithelial cells. In contrast, the highest titer in tracheal epithelial cells was observed at 72 hpi. (6.94 log_10_ TCID_50_/mL) (Fig. 4B). Swine H1N2 virus replicated the most efficiently in nasal turbinate epithelial cells, reached plateau at 48 hpi (8.27 log_10_ TCID_50_/mL), which is significantly higher than those achieved in other types of cells. Notably, it replicated less efficiently in mammary gland epithelial cells, with the highest titer (5.38 log_10_ TCID_50_/mL) detected at 72 hpi (Fig. 4C). Human H1N1 virus replicated well in nasal turbinate and lung epithelial cells, reaching plateau at 48 hpi, with titers of 8.17 log_10_ TCID_50_/mL and 8.40 log_10_ TCID_50_/mL, respectively, which are significantly higher than the titer obtained in mammary gland epithelial cells (5.50 log_10_ TCID_50_/mL). It replicated moderately well in trachea epithelial cells, with the highest titer (7.06 log_10_ TCID_50_/mL) achieved at 48 hpi (Fig. 4D).

Overall, bovine H5N1 replicates more rapidly and reached its plateau phase earlier than the other strains with particularly high replication in mammary gland epithelial cells. Primary nasal turbinate epithelial cells support the replication of all IAVs efficiently.

### Bovine H5N1 virus replication remains stable in immortalized porcine cells

Primary cells provide a relevant model to reflect the characteristics of their tissue of origin. However, primary cells have limited proliferation capacity and show proliferative arrest. Moreover, late-passage cultures show elevated basal expression of IFN-β, which can restrict host-virus interactions and replication (18). To set up a stable platform for investigating epithelial cell responses to IAV infections, we generated immortalized cell lines by transduction of the primary epithelial cells with SV40-T antigen followed by puromycin selection.

**Fig 5:**
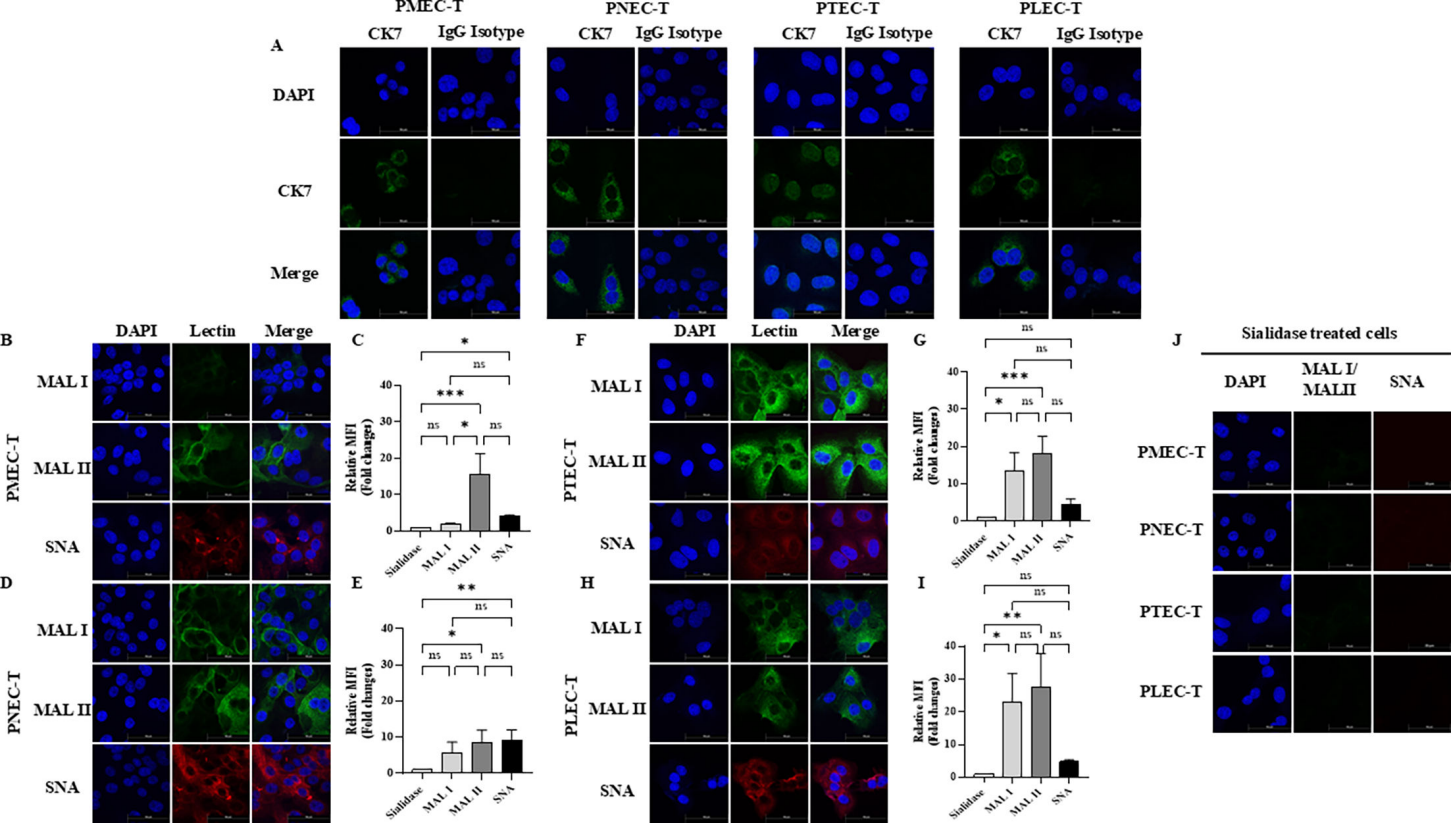
Expression of SA-α2,3 and SA-α2,6 receptors in immortalized porcine epithelial cells. Primary porcine epithelial cells were transduced with SV40-T antigen. (**A**). Identity of porcine immortalized epithelial cells from the mammary gland (PMEC-T), nasal turbinate (PNEC-T), trachea (PTEC-T), and lung (PLEC-T) was confirmed by detection of the epithelial cell marker cytokeratin-7. Porcine immortalized epithelial cells were stained with either monoclonal anti-CK7 or mouse IgG isotype, followed by staining with anti-mouse IgG H&L (Alexa Fluor 488, green) and DAPI (blue). SA-α2,3 and SA-α2,6 receptors of porcine immortalized mammary gland (**B**), nasal turbinate (**D**), trachea (**F**), and lung cells (**H**) were stained with biotinylated MAL-I (SA-α2,3-Gal-β1,4), MAL-II (SA-α2,3-Gal-β1,3), or SNA-Cy5 (SA-α2,6) (red). Cells stained with MAL-I or MAL-II were then stained with Streptavidin-Alexa Fluor 488 (green); subsequently, all slides were stained with DAPI (blue). Porcine immortalized epithelial cells were treated prior to lectin staining with sialidase for specific receptor staining (**J**). The lectin expression levels in porcine primary mammary gland (**C**), nasal turbinate (**E**), trachea (**G**), and lung cells (**I**) were quantified by MFI from three different areas of interest. Kruskal-Wallis test was used to compare the lectin levels. ns indicates non-significant; * indicates *P* < 0.05; ** indicates *P* < 0.01; *** indicates *P* < 0.005. Scale bar = 50 µm.

Immortalized porcine epithelial cells were characterized using an anti-cytokeratin 7 antibody to confirm the epithelial phenotype. Porcine immortalized cells from mammary gland, nasal turbinate, trachea, and lung expressed cytokeratin 7, consistent with their primary counterparts (Fig. 5A). To assess whether SA receptor profiles were altered during immortalization, we performed lectin-binding assays. Immortalized porcine mammary gland epithelial cells (PMEC-T) expressed significantly high levels of SA-α2,3-Gal-β1,3 (15.56-fold change), low levels of SA-α2,3-Gal-β1,4 (1.93-fold change), and moderate levels of SA-α2,6 (4.02-fold change) (Fig. 5B and C). Immortalized porcine nasal turbinate cells (PNEC-T) expressed SA-α2,3-Gal-β1,4 (5.79-fold change in MFI), SA-α2,3-Gal-β1,3 (8.38-fold change), and SA-α2,6 (9.07-fold change) (Fig. 5D and E). Immortalized porcine trachea epithelial cells (PTEC-T) expressed significantly high levels of SA-α2,3-Gal-β1,4 (13.46-fold change) and SA-α2,3-Gal-β1,3 (18.00-fold change). In contrast, SA-α2,6 was expressed at a moderate level (4.44-fold change) (Fig. 5F and G). In immortalized porcine lung epithelial cells (PLEC-T), similar patterns of SA expression profile were observed as with immortalized tracheal cells: SA-α2,3-Gal-β1,4 (22.94-fold change), SA-α2,3-Gal-β1,3 (27.55-fold change), and SA-α2,6 (4.83-fold change) (Fig. 5H and I). Sialidase-treated immortalized cells were stained negatively for MAL-I, MAL-II, and SNA (Fig. 5J). Although immortalization altered the receptor expression levels, as reflected by changes in MFI, compared with the corresponding primary cells, the overall patterns of avian and mammalian virus receptors in nasal turbinate and tracheal cells were maintained. The most noticeable change was observed in immortalized lung and mammary gland epithelial cells, which exhibited increased expression of SA-α2,3-Gal-β1,3 relative to their primary counterparts. Collectively, these results indicate that immortalization largely preserves epithelial cell identity while modulating SA receptor expression to a variable extent depending on the porcine cell type.

**Fig 6:**
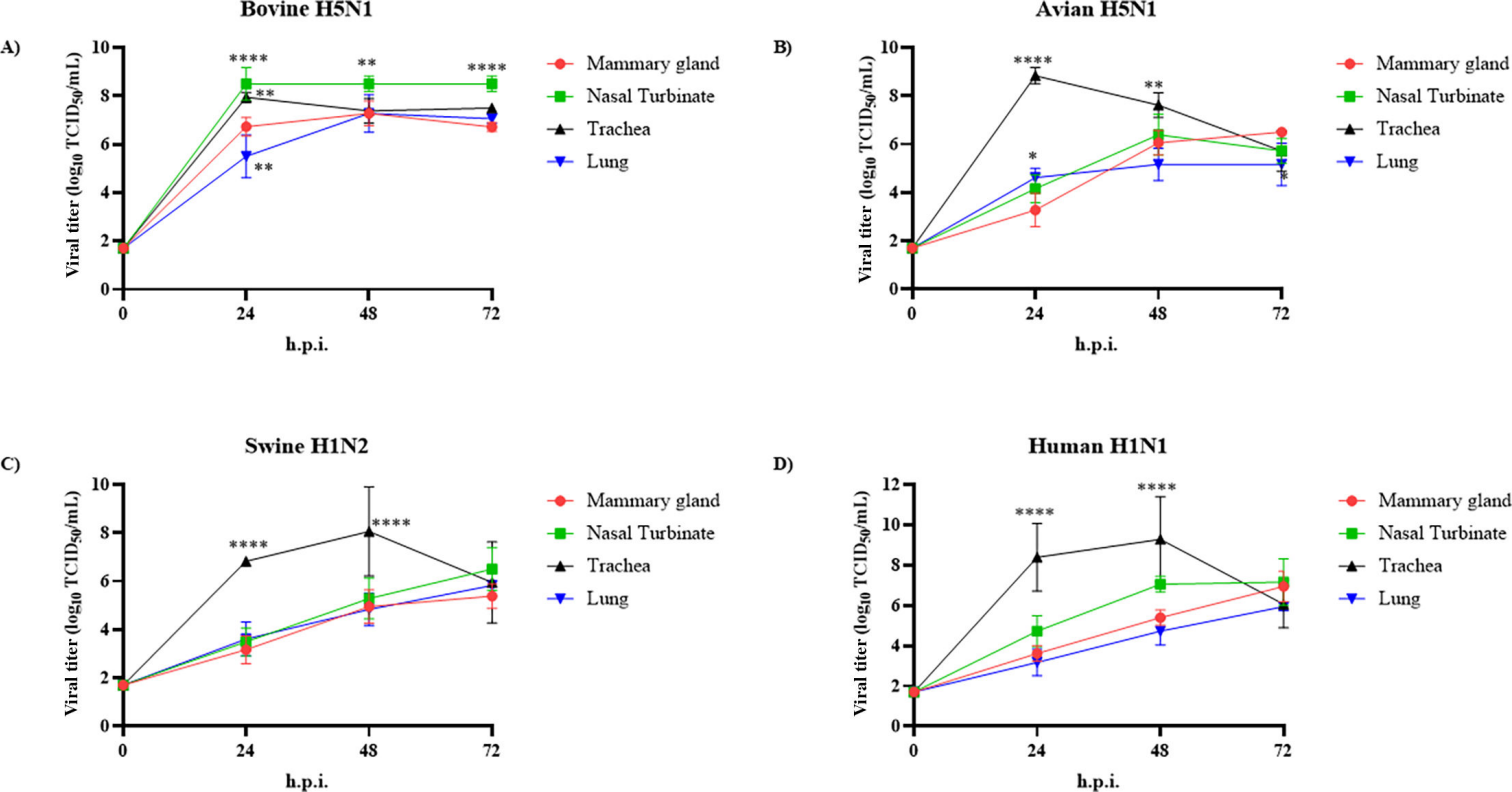
Replication kinetics of bovine, avian, swine, and human IAV in porcine immortalized epithelial cells. Porcine immortalized epithelial cells derived from the mammary gland, nasal turbinate, trachea, lung, and were infected with IAV bovine H5N1 (**A**), avian H5N1 (**B**), swine H1N2 (**C**), and human H1N1 (**D**) at MOI of 0.001. Supernatant was taken at 24, 48, and 72 hpi, and titrated on MDCK cells by TCID_50_ assay. Data show the mean of three independent experiments, and error bars indicate the standard deviation. Significance was calculated by two-way ANOVA; * indicates *P* < 0.05; ** indicates *P* < 0.01; **** indicates *P* < 0.001.

To assess the suitability of the immortalized porcine epithelial cells for supporting IAV replication, we performed infection assays and evaluated viral growth kinetics as was performed in primary cells (Fig. 6). Bovine H5N1 virus replicated efficiently in all porcine cell types, reaching a plateau at 24 hpi, except for lung epithelial cells. The highest viral titer (8.2 log_10_ TCID_50_/mL) was observed in immortalized nasal turbinate epithelial cells (Fig. 6A). These results are consistent with those observed in primary cells. Avian H5N1 virus replicated rapidly in immortalized trachea epithelial cells, achieving 8.8 log_10_ TCID_50_/mL at 24 hpi, which is significantly higher than the viral titers achieved in other cell types. The virus progressively replicated in lung, nasal turbinate, and mammary gland cells, reaching moderate titers by 72 hpi, ranging from 5.7 to −6.5 log_10_ TCID_50_/mL (Fig. 6B). Similar replication patterns were observed for swine H1N2 virus and human H1N1 virus in immortalized cells (Fig. 6C and D). Both viruses replicated robustly in trachea cells and reached peak titers at 48 hpi, with 8.1 log_10_ TCID_50_/mL and 9.3 log_10_ TCID_50_/mL, respectively. While swine H1N2 virus replicated steadily and reached titers in the range of 5.3–6.5 log_10_ TCID_50_/mL in mammary gland, lung, and nasal turbinate cells at 72 hpi, human H1N1 virus replicated slightly more efficiently in these types of cells, reaching titers in the range of 6.06–7.17 log_10_ TCID_50_/mL.

Overall, bovine H5N1 maintained consistent replication across all immortalized cell types. Conversely, avian H5N1, swine H1N2, and human H1N1 exhibited reduced viral titer in immortalized nasal turbinate and lung epithelial cells and increased viral titer in immortalized trachea epithelial cells. Immortalized mammary gland cells supported similar replication levels for all viral strains, except human H1N1, whose titer was elevated approximately 1 log_10_.

## DISCUSSION

The recent report of bovine mammary gland infection by HPAI H5N1 clade 2.3.4.4b (19, 20) challenges the classical paradigm of IAV tissue tropism, since the respiratory tract has been considered the principal site of IAV infection. In this study, we investigated the susceptibility of the porcine mammary gland tissues and cells to IAVs of different species origin.

We show that the porcine mammary gland tissue, nasal turbinate, bronchiole, and alveoli express both SA-α2,3 and SA-α2,6 receptors, suggesting that these tissues may be susceptible to attachment by both avian and mammalian origin IAVs. In contrast, porcine trachea tissue expressed SA-α2,3-Gal-β1,4 and SA-α2,6 receptors, suggesting that it could be more susceptible to binding of IAV of wild birds and mammalian origin, with limited binding potential for avian-origin strains that preferentially recognize SA-α2,3-Gal-β1,3 receptors typically associated with poultry birds. The results obtained in respiratory tract tissues are consistent with those reported by Kristensen et al. (13), but differ slightly from the results reported by Nelli et al. (9), who showed similar expression of SA-α2,3 and SA-α2,6 in alveoli, while we observed significantly higher expression of SA-α2,6 compared with SA-α2,3-Gal-β1,4 and SA-α2,3-Gal-β1,3. These differences may reflect inherent biological variability among samples. The three studies were conducted on pigs of different ages and sexes: Kristensen et al. analyzed tissues from 8- to 9-week-old piglets, Nelli et al. used post-weaned male pigs aged 4–8 weeks, and our study analyzed tissues from a lactating sow. It is important to note that, despite minor variations in sialic acid receptor expression, both α2,6- and α2,3-linked sialic acid receptors are consistently expressed throughout the respiratory tract, supporting the idea that pigs serve as a mixing vessel for influenza viruses. Notably, we showed the presence of both SA-α2,3 and SA-α2,6 receptors in porcine mammary gland, highlighting a previously underappreciated tissue niche that may serve as a novel binding target for diverse IAV subtypes.

Results from the viral tissue-binding assays showed that bovine H5N1, avian H5N1, and swine H1N2 viruses could bind to all tissues tested, though with differing intensities. In trachea, SA receptor expression was low (Fig. 1E and F), yet binding to avian H5N1 and swine H1N2 viruses was strong. Most binding was observed mainly in the epithelial lining where mucus covers the epithelium (Fig. 2E and F). The discrepancy between lectin staining and viral binding may be explained by the presence of sialylated glycans on secreted mucins, which are not strictly recognized by plant lectins (21), or simply because lectin staining was weak. We acknowledged that in our study, porcine tissues are from a single donor. Inter-individual variation in glycan expression and viral replication efficiency in porcine epithelial cells have been reported between donors (22). While lectin immunofluorescence provides excellent spatial resolution, lectin binding and virus binding affinity can vary with tissue fixation and processing conditions.

We isolated porcine epithelial cells from the same tissues and used both primary and immortalized cells to evaluate the expression of SA-α2,3 and SA-α2,6 receptors and their capacity to support viral replication. The expression of SA receptors in mammary gland cells was consistent with those detected in corresponding tissue. Nasal turbinate, trachea, and lung epithelial cells showed differences when compared with tissues of the same origin. While nasal turbinate tissue displayed moderate expression levels of SA-α2,3-Gal-β1,3, SA-α2,3-Gal-β1,4, and SA-α2,6 receptors, primary nasal turbinate epithelial cells displayed elevated levels of these receptors, especially for SA-α2,6. In the trachea, the expression is contrasting, while SA receptors in trachea tissues were weak, the display of SA receptors in primary trachea epithelial cells was elevated, with the expression of SA-α2,3-Gal-β1,4 being the highest. Lung tissues showed moderate expression of SA receptors; however, primary lung epithelial cells expressed elevated SA receptors, with the expression of SA-α2,3-Gal-β1,4 being the highest. This divergence could be explained by primary cells being a homogeneous population of a single cell type, while whole tissues maintain cellular architecture, having multiple cell types including extracellular matrix and dense tissue that can hinder lectin accessibility.

All primary cell types supported the robust replication of bovine H5N1 virus. Notably, although mammary gland epithelial cells expressed relatively high levels of SA-α2,6, they did not support replication of swine H1N2 or human H1N1 viruses to high titers (≤6 log_10_/mL). Furthermore, although greater binding of avian H5N1 virus to mammary gland tissue was observed compared with bovine H5N1 virus, avian H5N1 replicated to lower titers than bovine H5N1 in mammary gland epithelial cells at nearly all time points. Collectively, these results suggest that receptor abundance and virus-receptor binding are not sufficient to determine viral replication capacity; rather, they provide a prerequisite for viral replication within a given tissue. Efficient viral replication further depends on additional viral and host factors, including viral RNA-dependent RNA polymerase activity, which is constrained by host factor ANP32 (23). In addition, NS1 and PA-X mediate immune evasion, suppressing host antiviral responses and promoting viral replication (24).

Bovine H5N1 replicated well in porcine primary epithelial cells derived from trachea; however, the virus did not bind to the tissues well. These results may suggest that minimal binding is sufficient for viral replication in porcine cells, or it is also possible that the weak receptor binding observed was due to the limitation of virus detection in binding assays or limitation of the immunostaining assay. Similar results were observed in goats that were inoculated intranasally with both bovine H5N1 virus and an avian H5N1 virus, where limited or undetectable viral replication was observed in the lung tissues; however, robust viral replication was observed in goat primary cells isolated from the nasal turbinate, bronchioles, and alveoli (25).

Notably, we observed immortalized trachea cells support avian H5N1, swine H1N2, and human H1N1 viruses’ replication to 2–3 log higher titers than primary counterparts at 24 or 48 hpi. In line with our findings, a previous study with immortalized porcine trachea epithelial cells showed viral titers can reach up to 8.5 log_10_ TCID_50_/mL with human H1N1 virus infection, which was higher than in MDCK cells (26). Our analysis of SA receptors showed that after immortalization, the levels of SA-α2,3 and SA-α2,6 receptors were slightly reduced, suggesting that the increased viral titer was not due to an increase of the SA. It was reported that immortalization of porcine tracheal epithelial cells increases viral titres and reduces IFN-β expression following swine H1N1 infection (27). Although we did not assess innate immune responses in this study, this represents an important avenue for future investigation.

Experimental infection of pigs with avian H5N1 clades 1, 2.1, 2.2, and 2.3 have shown limited replication with either mild symptoms or animals being asymptomatic (28). In other studies, pigs infected with H5N1 clade 2.3.4.4 or 2.3.4.4b showed limited viral replication and no transmission occurred to directly contacted naïve pigs (29, 30). Recently, a study with bovine-derived HPAI H5N1 genotype B3.13 in pigs demonstrated limited replication in the upper respiratory tract (31). Our *in vitro* results showed that both porcine respiratory epithelial cells and mammary epithelial cells supported replication of IAVs from bovine, avian, swine, and human origins, with the bovine HPAI H5N1 isolate replicating particularly efficiently in mammary cells. This discrepancy underscores a well-recognized difference between *in vitro* cell models and whole animal infection outcomes: cells in culture often reveal the intrinsic ability of a virus to bind to, enter, and replicate in cells; *in vivo* infection is determined by additional physiological barriers, including mucus barrier, tissue architecture, receptor accessibility, viral fitness, and host innate immune defenses, etc. Notably, our findings expand the recognized tissue tropism of IAVs in swine, revealing that the mammary gland may serve as an alternative and previously overlooked replication site for both avian and mammalian origin viruses. Given the increasing frequency of interspecies spillover events and the expanding host range of HPAI H5N1 viruses, further investigation of non-respiratory tissues, particularly the mammary gland, in animal experiments is essential for assessing the zoonotic and agricultural risks posed by emerging IAVs.

## MATERIALS AND METHODS

### Biosafety

Work with HPAI H5N1 was carried out in Vaccine and Infectious Disease Organization (VIDO) containment level 3 (CL3) facility, in compliance with the Canadian Biosafety Standard, 3rd edition, under the authority of the Public Health Agency of Canada and the Canadian Food Inspection Agency.

### Cells and viruses

Madin-Darby canine kidney (MDCK) (ATCC, #CRL-2936) cells were maintained in Minimal Essential Medium (MEM) (Sigma-Aldrich, M4655) supplemented with 10% fetal bovine serum (FBS) (Corning, 35-010-CV), and maintained in a humidified 5% CO_2_ incubator at 37°C. A/dairy cattle/Texas/24-008749-002/2024 (H5N1) (bovine H5N1), A/Turkey/Ontario/6213/66 (H5N1) (avian H5N1), A/Swine/Alberta/SD0191/2016 (H1N2) (swine H1N2), and A/Halifax/210/2009 (H1N1) (human H1N1) were propagated in MDCK cells in viral growth media (MEM containing 1 μg/mL L-1-tosylamide-2-phenylethyl chloromethyl ketone [TPCK] treated trypsin [Affymetrix, 22725-250MG] and 0.2% bovine serum albumin [BSA] [Sigma-Aldrich, A7030]). For IAV binding assay, all viruses were inactivated. Avian H5N1 and swine H1N2 were inactivated by ultraviolet light exposure for 30 min. Bovine H5N1 was inactivated by treatment with 97% β-propiolactone (Thermo Fisher Scientific, AAB2319703) to a final concentration of 0.1%. The mixture was rocked at 4°C overnight, followed by incubation at 37°C for 2 h to allow hydrolysis of β-propiolactone, as described previously (32).

### Tissue collection and preparation

VIDO veterinary services obtained mammary gland, alveoli, bronchiole, trachea, and nasal turbinate tissues from a non-infected sow. Tissues were maintained in 10% neutral-buffered formalin, then embedded in paraffin, cut into 4-μm sections, and mounted onto glass slides.

Paraffin-embedded tissue sections were deparaffinized by immersion in xylene for 10 min, followed by sequential rehydration using ethanol solutions (100%, 95%, 75%, 50%, 0%) for 5 min each. Sections were then heated in 50 mM sodium citrate at 95°C for 10 min to restore SA receptor accessibility, then rinsed with distilled water.

### Lectin staining

Tissue sections with SA receptor-restored or porcine epithelial cells were incubated with 5% bovine serum albumin (BSA, Sigma-Aldrich, A7030) in phosphate-buffered saline (PBS) for 1 h at room temperature (RT) and washed four times with PBS. SNA-Biotinylated (2 μg/mL, Vector Laboratories, B-1305-2) or SNA-Cy5 (10 μg/mL, Vector Laboratories, CL-1305-1), MAL-I-Biotinylated (2 μg/mL, Vector Laboratories, B-1315-2) and MAL-II-Biotinylated (2 μg/mL, Vector Laboratories, B-1265-1) were added to the slides and incubated for 1 h at RT, followed by four 5-min washes with PBS. Sections stained with biotinylated lectins were incubated with Streptavidin-Alexa 488 (1 μg/mL, Thermo Fisher Scientific, S32354) for 1 h at RT. Subsequently, tissue sections or porcine epithelial cells were washed four times with PBS for 5 min each. Finally, all samples were incubated with DAPI (Thermo Fisher Scientific, 62248) for 5 min at RT for nuclei staining, followed by four washes with PBS for 5 min and rinsed with distilled water. To rule out non-specific binding of lectins, tissue sections or porcine epithelial cells were treated, prior to lectin staining, with 50 units of sialidase A (New England Biolabs, P0722S) for 1 h at 37°C. Stained sections or cells were mounted with Prolong gold antifade Mountant (Thermo Fisher Scientific, P36934) and visualized using Leica SP8 confocal microscope.

### Immunofluorescence IAV staining

Inactivated IAVs were purified by gradient sucrose ultracentrifugation and resuspended in TSE buffer (0.02 M Tris, 0.015 M NaCl, and 0.002 M EDTA). Total protein concentration was measured by absorbance at 280 nm. Tissue sections with SA-receptor-restored were incubated with 5% BSA (Sigma-Aldrich, A7030) in PBS for 1 h at RT, followed by four washes with PBS. Sections were then incubated individually with inactivated bovine H5N1, avian H5N1, or swine H1N2 at a concentration of 100 μg/mL at 4°C overnight. Slides were washed four times with PBS, and fixed with 4% paraformaldehyde (PFA) (Sigma, P6148) for 10 min at RT. Then, sections were incubated with 5% Triton X-100 (Sigma) for 5 min at RT, followed by four 5-min washes with PBS. Sections were incubated with rabbit anti-M1 polyclonal antibody (in-house) at 1:1,000 dilution for 2 h at RT, followed by four 5-min washes with PBS. After washing steps, all slides were incubated with Alexa Fluor 488-conjugated anti-rabbit secondary antibody (1:5,000, Thermo Fisher Scientific, A-11008) for 30 min at RT, followed by four, 5 min washes with PBS. Finally, tissue sections were incubated with DAPI (Thermo Fisher Scientific, 62248) for 5 min at RT to visualize nuclei. To rule out non-specific binding of IAV, tissue sections were treated, prior to IAV binding, with 50 units of sialidase A (New England Biolabs, P0722S) for 2 h at 37°C. Stained sections were mounted with Prolong Gold antifade mountant and visualized using Leica SP8 confocal microscope.

### Primary cell isolation

The nasal turbinate, trachea, lung (alveolar section), and mammary gland were obtained from a humanely euthanized sow. Tissues were placed in sterile ice-cold PBS containing 50 μg/mL gentamicin (GIBCO, 15750060) and 1× penicillin-streptomycin-neomycin [(PSN) Thermo Fisher Scientific, 15640055]. All tissues were washed two times with ice-cold PBS containing 50 μg/mL gentamicin and 1× PSN. Trachea and nasal turbinate were cut longitudinally, and the submucosal layer was removed using a scalpel. All tissues were cut into 1–2 cm^3^ pieces and then minced into approximately 5 mm^3^. Minced tissues were incubated with DMEM/F12 (Thermo Fisher Scientific, 12634010) supplemented with 5% FBS (Sigma), 1 mg/mL collagenase A (MilliporeSigma, G1397), 0.05% hyaluronidase (Millipore-Sigma, H3506), 50 μg/mL gentamicin in 1× PSN for 12 h at 37°C with gentle agitation. Digested tissues were filtered using a 70-μm cell strainer, the strainer was rinsed with DMEM/F12, and cells were centrifuged at 800 × *g* for 3 min at 25°C. Cell pellets were washed three times with PBS containing 1× PSN.

Cell pellets were resuspended with DMEM/F12 (Thermo Fisher Scientific, 12634010) supplemented with 10% FBS (Sigma), 5 μg/mL insulin (GIBCO, 12585014), 1 μg/mL hydrocortisone (Stemcell Technologies, 07926), 5 ng/mL epidermal growth factor (EGF, GIBCO, PHG0315), 50 μg/mL gentamicin, and 1× PSN. Primary cells were seeded in standard T25 flasks at 1 × 10^7^ cells/flask and incubated 16 h in a humidified 5% CO_2_ incubator at 37°C. Non-adherent cells were transferred to a T25 flask (Sarstedt, 83.39.302) to challenge adherent cells and to obtain primary cell cultures. Epithelial cells adhered after 24-48 h, depending on the cell type. The epithelial cells were cultured in 5% CO_2_ incubator at 37°C for 5–8 days until they reached 100% confluence. The culture media were replaced on days 3 and 6. Cells were used for infection between passages 4–10.

### Immortalization of porcine primary cells

Porcine primary cells were transduced with lentivirus expressing simian virus 40 (SV40) T-antigen, as described previously (33). Briefly, a 70% confluent T25 flask of the porcine primary cells was trypsinized and resuspended in 5-mL media. Then, 200 μL of cell suspension was transferred to a sterile 1.5-mL microcentrifuge tube, mixed with 200 μL of the SV40 T-antigen encoding lentivirus, and 3.6 µg Polybrene (EMD Millipore; TR-1003-G) was added to this mix. The resulting 400-μL mix was carefully dispensed into a six-well plate. Cells were incubated with the virus for 1 h at 37°C, following which 2 mL of complete media was added to the well. Media were changed after 24 h of infection. Once the cells were confluent, they were trypsinized and transferred to a T25 flask. Twenty-four hours post-transfer, media on the cells were changed with media containing 2 μg/mL puromycin to select for stably transduced cells.

### Immunofluorescent cytokeratin-7 staining and confocal microscopy

Porcine primary and immortalized cells were seeded in eight-well chambers at 3 × 10^4^ cells/well and cultured overnight in a humidified 5% CO_2_ incubator at 37°C. Primary cells were fixed in 1:1 v/v methanol (VWR, BDH1135-4LP): acetone (Sigma, 534064-500ML) at −20°C for 20 min, then cells were incubated with 5% Triton X-100 (Sigma) for 5 min at RT, followed by four 5-min washes with PBS. Cells were blocked in 5% BSA (Sigma) for 1 h at RT, followed by four 5-min washes with PBS. Cells were incubated with mouse anti-cytokeratin-7 (CK7; 10 μg/mL; Thermo Fisher Scientific, MSM14-949-P0) for 2 h at RT, followed by four 5-min washes with PBS. Then, cells were incubated with Alexa Fluor-488 conjugated anti-mouse IgG (1:1000; Invitrogen; A10680) for 30 min at RT. Finally, cells were incubated with DAPI (Thermo Fisher Scientific, 62248) for 5 min at RT to visualize nuclei, followed by four 5-min washes with PBS and rinsed with distilled water. To corroborate specific binding of anti-CK7, cells were incubated with mouse IgG isotype (Cell Signaling, 5415S) instead of anti-CK7 as a control. Slides were mounted with Prolong Gold antifade mount and visualized using Leica SP8 confocal microscope.

### Viral growth kinetics

Porcine primary or immortalized cells were seeded in 12-well plates at 5 × 10^5^ cells/well, and infected with bovine H5N1, avian H5N1, swine H1N2, or human H1N1 at MOI of 0.001. Viruses were incubated 1 h in a humidified 5% CO_2_ incubator at 37°C. Then, inoculum was removed, and viral growth medium was added containing 0.5 (for primary cells) or 1 μg/mL (for immortalized cells) TPCK/trypsin. Supernatant samples were collected at 24-h intervals until 72 h. The viral titer was determined by TCID_50_, as described previously (34).

### Image analysis

Quantitative analysis of lectin and IAV binding in porcine tissues and cells was performed using Leica LAS X software. The mean fluorescence intensity (MFI) of lectin staining or virus binding was first normalized to the corresponding DAPI MFI. The MFI in sialidase-treated slides was calculated using the same normalization procedure. Finally, the normalized MFI from non-treated samples was divided by the normalized MFI from the corresponding sialidase-treated samples to obtain relative MFI and is expressed as fold change. For each slide, MFI measurements were taken from three areas of interest.

### Statistical analysis

The statistical analysis was performed in GraphPad Prism 11.0.0 (GraphPad Software, Inc., San Diego, CA, USA) using one-way ANOVA and Kruskal-Wallis test for image analysis and two-way ANOVA for replication kinetics. Data are shown as means ± SD. Significant differences are denoted by *P < 0.05, **P < 0.01, ***P < 0.001, or ****P < 0.0001.

## Data Availability

All data are fully available without restriction.
